# A Two Joint Neck Model to Identify Malposition of the Head Relative to the Thorax

**DOI:** 10.3390/s21093297

**Published:** 2021-05-10

**Authors:** Philipp M. Schmid, Christoph M. Bauer, Markus J. Ernst, Bettina Sommer, Lars Lünenburger, Martin Weisenhorn

**Affiliations:** 1 Institute of Signal Processing and Wireless Communications, School of Engineering, Zurich University of Applied Sciences, Technikumstrasse 9, 8400 Winterthur, Switzerland; scdp@zhaw.ch (P.M.S.); weie@zhaw.ch (M.W.); 2 Institute of Physiotherapy, School of Health Professions, Zurich University of Applied Sciences, Katharina-Sulzer-Platz 9, 8400 Winterthur, Switzerland; erus@zhaw.ch (M.J.E.); somb@zhaw.ch (B.S.); 3 Hocoma AG, Industriestrasse 4, 8604 Volketswil, Switzerland; lars.luenenburger@dih.com

**Keywords:** neck, protraction, posture monitoring, neck pain, stereophotogrammetry, biomechanical model, movement analysis

## Abstract

Neck pain is a frequent health complaint. Prolonged protracted malpositions of the head are associated with neck pain and headaches and could be prevented using biofeedback systems. A practical biofeedback system to detect malpositions should be realized with a simple measurement setup. To achieve this, a simple biomechanical model representing head orientation and translation relative to the thorax is introduced. To identify the parameters of this model, anthropometric data were acquired from eight healthy volunteers. In this work we determine (i) the accuracy of the proposed model when the neck length is known, (ii) the dependency of the neck length on the body height, and (iii) the impact of a wrong neck length on the models accuracy. The resulting model is able to describe the motion of the head with a maximum uncertainty of 5 mm only. To achieve this high accuracy the effective neck length must be known a priory. If however, this parameter is assumed to be a linear function of the palpable neck length, the measurement error increases. Still, the resulting accuracy can be sufficient to identify and monitor a protracted malposition of the head relative to the thorax.

## 1. Introduction

### 1.1. Neck Pain

Neck pain is a frequent health complaint and is globally ranked in third place for musculoskeletal disorders with a yearly prevalence of more than 15 percent in 2013, which has risen by 54 percent since 1990 [1]. In western countries, office workers in particular have been found vulnerable to develop neck pain [2]. Neck pain symptomatic female office workers showed a protraction of the head relative to the thorax, also known as a protracted “forward head posture” (FHP), compared to asymptomatic controls [3]. A FHP has frequently been reported for multiple activities and postures, such as driving, computer work, or reading [4]. It has furthermore strongly been associated with headaches such as migraines, tension-type, and cervicogenic headache [5,6], neck pain, and even shoulder problems [7,8,9,10]. Additionally FHP is associated with impairments related to decreased neck proprioception such as balance [11,12,13] and visual impairments [14]. Cervical range of motion (ROM) has been found reduced when performed out of a FHP [15] and altered for coupled movements [16]. Park et al. found reduced upper cervical (UCS) flexion ROM due to sustained computer work [17] and Ernst et al. demonstrated strong associations between reduced UCS flexion and headache [18]. Protraction and retraction are regarded as combined and opposite movements of the upper cervical (UCS = occiput to cervical vertebra 3), and the lower cervical-spine (LCS = cervical vertebrae 3–7) [19]. Besides office workers, adolescents are regarded especially susceptible to adopt FHP, particularly when frequently using unfitted computers or other technology [7,20,21,22]. Therefore, monitoring the occurrence of a FHP while working or gaming on a computer screen might be regarded useful to prevent those aforementioned impairments. However, the evidence regarding the validity of clinical techniques to determine an exact craniovertebral posture is inconsistent and only little evidence for the assessment of cervical ROM is provided [23]. One difficulty is that the cervical spine moves around 3-D axes [19]. Therefore, different initial positions will lead to different axial rotation values and these axial rotation values also differ between younger and older, female and male persons [24].

### 1.2. Identifying Malposition

A prolonged malposition of the head can be prevented through feedback [25]. However, to provide feedback, a measuring device must allow for posture tracking. Even though modern marker-based 3-D motion capture systems are capable of tracking position and rotation accurately in six degrees of freedom [26], they are either time-consuming, expensive, stationary, or all of the above and therefore are inappropriate for daily use. However, other approaches to measure FHP already exist. Instead of measuring the displacement of the head [27,28], the craniovertebral angle (CVA) method is widely used to assess FHP [17,29,30,31]. Pürkhauer et al. used the Kinect^®^ face tracking algorithm to measure FHP [32]. Another approach is to use a combination of inertial measurement units (IMU) and strain gauges on the neck, which allows tracking the neck angle [33]. Even though these approaches seem promising, economic measurement systems are needed for reliable, valid, and objective ongoing monitoring, for example during computer work or gaming. Existing IMU models measure the angles of the neck accurately to some extent, but none of these models include an estimate of translation which would be important for the study of forward head postures [34,35,36]. In contrast, camera-based systems limit a persons’ range of motion because the person must always act within the field of view of the cameras [32,34,37]. A simple biomechanical model of the neck that can estimate translation but is independent of cameras would therefore be of great advantage. In clinical practice, it would be preferable to use an already existing measurement system, like IMU or electromagentic tracking systems [38,39,40,41]. However, first a precise biomechanical model is needed, which ideally is developed with the measurement data of a high-precision measurement system, such as a 3-D optoelectronic system [42,43,44,45].

### 1.3. Contribution

To overcome these limitations, this study proposes a simple neck model to explain head orientation and translation relative to the thorax. Due to its simplicity, only two rigid bodies are required to infer the relative position of the head with respect to the thorax, a thorax, and a neck stick. An important parameter in this model is specifically the length of the neck stick. In this work we determine (i) the accuracy of the proposed model when the neck length is known, (ii) the dependency of the neck length on the body height, and (iii) the impact of an incorrect neck length on the models accuracy.

## 2. Neck Model

**Figure 1:**
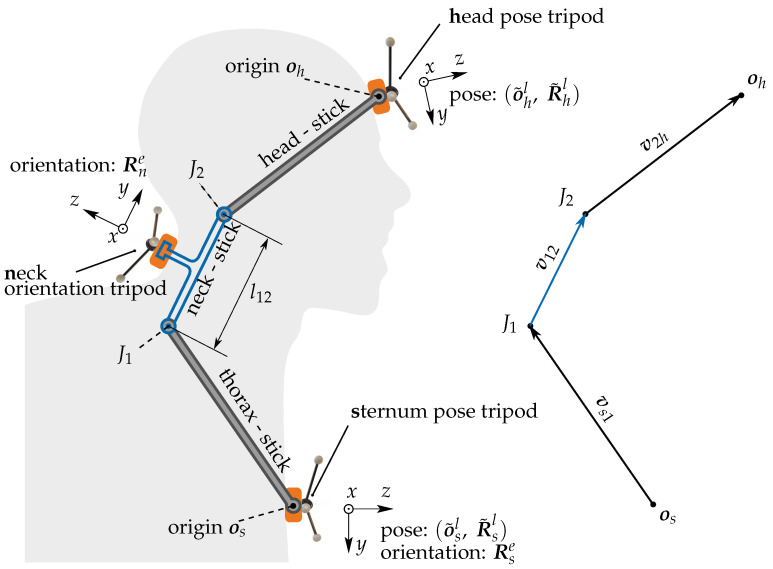
The neck model shown in blue contains the neck-stick which is assumed to be rigidly connected to the neck rigid body and the sternum rigid body. The extended model comprises one pose sensing tripod at the sternum and one at the forehead. These two tripods are assumed to be rigidly connected to head-stick and the thorax-stick. The joints $J_{1}$ and $J_{2}$ are ball joints. The extended model, comprising a head-stick and head rigid body is introduced for identification of the neck-stick $l_{12}$.

The cervical spine has the largest mobility of all sectors of the human spine. Since the head moves with six degrees of freedom relative to the thorax, the cervical spine has six degrees of freedom [17,37,46]. The range of motion differs between directions. In particular, along the spinal direction, motion is limited to few millimeters only. Therefore, a simplified model with five degrees of freedom could be used to describe relative head motion with an accuracy that is satisfactory for clinical applications such as biofeedback on FHP. In this work we propose a simplified neck model consisting of two ball joints, one in the proximity of vertebra C2 and the other in the proximity of vertebra C7. The two ball joints $J_{1}$ and $J_{2}$ are connected with a stiff neck-stick of fixed length $l_{12}$ (Figure 1). Each ball joint offers three rotational degrees of freedom. However, one of them, i.e., the rotation around the neck-stick axis, is common to both joints such that head motion is modeled by only five degrees of freedom. In this model the position of the head center is defined to be the position of $J_{2}$. The such defined head position does not depend on rotations of the head around $J_{2}$. Furthermore, the relative head position is defined as the position of $J_{2}$ relative to $J_{1}$ and is described by the vector $l_{12}$ rotated according to the orientation measurement of the neck.

**Figure 2:**
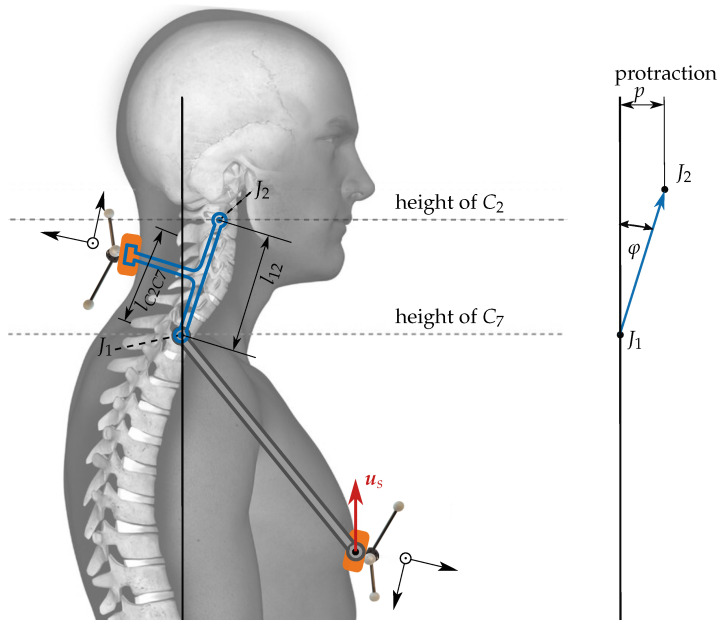
Determination of the protraction *p* from the orientation of the neck relative to the orientation of the thorax. The red arrow is fixed to the thorax tripod. It is defined to be exactly vertical when the upright posture is assumed during the calibration phase. The distance between C2 and C7, measurable by palpation is denoted as $l_{C2C7}$.

Clinical assessments do not allow for reference measurements of the neck length and thus prohibit identification of the exact neck length for an individual. To overcome this limitation, the neck-stick length $l_{12}$ could be predicted from an individual’s body size assuming that a taller person has a longer neck. An even more precise predictor for $l_{12}$ should be the measurable distance $l_{C2C7}$ between the vertebra C2 and C7 (Figure 2). As we expect $l_{12}$ to grow with $l_{C2C7}$ and for simplicity we assume a linear model:

(1) $$l_{12}=\beta_{1}\cdot l_{C2C7}+\beta_{0},$$

with offset $\beta_{0}$ and sensitivity $\beta_{1}$. The quality of this prediction will be discussed in Section 4.1. The purpose of the neck model is to describe the 3-D position of the head relative to the thorax, which is equivalent to describing the position of joint $J_{2}$ relative to joint $J_{1}$. The compelling feature of this model is that the position of the head center $J_{2}$ is determined by just two easily measurable orientations, the orientation of the thorax- and the orientation of the neck-stick. The measurement of these orientations in local earth coordinates could later on be conducted with economic instrumentation such as IMUs that include a magnetometer and sensor fusion.

### 2.1. Coordinate Frames

To enable numeric computations, vectors are expressed with respect to a specific coordinate frame, for simplicity called frame. A specific frame *b* is defined by an orientation $R_{b}$ and an origin $o_{b}$ i.e., by the tuple $\left(o_{b},R_{b}\right)$. We define both, the origin and the orientation with respect to some other frame, say frame-*g*. To indicate this, we set *g* as a superscript and form the notation $\left(o_{b}^{g},R_{b}^{g}\right)$ to denote the frame-*b* which is expressed in terms of frame-*g*. The concept of a frame is identical to the concept of a pose. Therefore, the terms frame and pose are used synonymously. In this work we define four different frames: (i) The earth-frame *e*, whose orientation is defined by the local gravitation and the magnetic field vector and whose origin is any arbitrary position. (ii) The local frame *l*, defined by the optoelectronic measurement equipment, (iii) the sternum frame *s*, and (iv) the head frame *h*. A vector $v^{e}$ indicating a specific position with respect to frame-*e*, is related to the vector $v^{l}$ that indicates the same position with respect to frame-*l* by the following equation:

(2) $$v^{e}=R_{l}^{e}\cdot v^{l}-o_{l}^{e}.$$

In contrast, if the vector $v^{l}$ indicates a specific direction, the origin $o_{l}^{e}$ must be omitted. In this notation we can write $R_{e}^{l}$ for the matrix inverse of $R_{l}^{e}$.

### 2.2. Model Parameter Identification

The proposed model contains the length $l_{12}$ of the neck-stick as its only parameter. This length is an a priory unknown as the joints $J_{1}$ and $J_{2}$ are not defined by palpable anatomical landmarks. However, the direction of the neck-stick is identical to the *y*-axis of the rotation $R_{n}^{e}$ of the neck rigid body. To enable identification of $l_{12}$, we extend the setup by two rigid segments. A head segment located on the forehead, and a sternum segment placed on the sternum with their respective poses $\left(o_{h}^{l},\;R_{h}^{l}\right)$ and $\left(o_{s}^{l},\;R_{s}^{l}\right)$. The unknown vector pointing from the origin $o_{s}$ to the center of joint $J_{1}$ is called the thorax vector $v_{s1}^{s}$. Another unknown is the head vector $v_{2h}^{h}$ pointing from $J_{2}$ to the origin $o_{h}$ of the head segment. These two vectors are assumed to have constant entries under head motion, when they are expressed with respect to their native frames *s* and *h*, respectively. In total, the extended model contains seven unknown constants. They can be identified by fitting the predicted to the measured head position.

#### 2.2.1. Predicted Head Position

The prediction ${\hat{o}}_{h}$ of the head’s origin $o_{h}$ can be expressed with respect to the *s*-frame according to Figure 1:

(3) $${\hat{o}}_{h}^{s}=v_{s1}^{s}+v_{12}^{s}+v_{2h}^{s}.$$

The direction of the neck-stick vector $v_{12}^{e}$ is determined by the *y*- axis of the orientation matrix $R_{s}^{e}$, which is provided by the neck rigid body, i.e., $v_{12}^{e}=l_{12}\cdot {\left(0,1,0\right)}^{T}$ and $v_{12}^{s}=R_{e}^{s}\cdot v_{12}^{e}$. Furthermore, by using the identity $v_{2h}^{s}=R_{h}^{s}\cdot v_{2h}^{h}$, we can express the predictor (3) as

(4) $${\hat{o}}_{h}^{s}=v_{s1}^{s}+R_{e}^{s}\cdot l_{12}\cdot {\left(0,1,0\right)}^{T}+R_{h}^{s}\cdot v_{2h}^{h}.$$

#### 2.2.2. Measured Head Position

The head position $o_{h}$ can be inferred from the pose measurements of the head and sternum:

(5) $$o_{h}^{s}=R_{l}^{s}\cdot \left(o_{h}^{l}-o_{s}^{l}\right).$$

#### 2.2.3. Parameter Fitting

The desired parameter is the neck-stick length $l_{12}$, while the thorax vector $v_{s1}^{s}$ and the head vector $v_{2h}^{h}$ constitute six scalar nuisance parameters. These seven parameters minimize the sum of squared Euclidean errors between prediction ${\hat{o}}_{h}^{s}$ and measurement ${\tilde{o}}_{h}^{s}$ as:

(6) $$l_{12},\;v_{s1}^{s},\;v_{2h}^{h}=\underset{l_{12},\;v_{s1}^{s},\;v_{2h}^{h}}{\mathrm{argmin}}\sum_{i\in I}\sum_{t\in T_{i}}{∥{\hat{o}}_{h}^{s}\left(i,t\right)-{\tilde{o}}_{h}^{s}\left(i,t\right)∥}_{2}^{2},$$

where I is the set of all measurements of one participant and $T_{i}$ is the set of sampling time instants *t* in the measurement *i*. The difference ${\hat{o}}_{h}^{s}-{\tilde{o}}_{h}^{s}$ linearly depends on these parameters. Hence, the optimization problem at hand is a linear least squares problem that can be solved in a single step [47].

### 2.3. Calibration Procedure

Assuming that rigid bodies have an ideal accuracy, the orientations of the rigid bodies still need to be adjusted, so that the sensed rotation matrices equal:

$$R_{n}^{e}=R_{s}^{e}=I_{3},$$

where $I_{3}$ is the unity matrix with dimension 3×3, at calibration time $t=t_{0}$, when an upright posture is assumed by the participant.

### 2.4. Measuring Protraction

The described neck model determines rotation but also translation, e.g., protraction of the head relative to the thorax. Figure 2 shows a moderate protraction *p* in the sagital domain. The protraction angle φ results in the head displacement or protraction $p=l_{12}\;\sin\left(\varphi \right)$. A vertical alignment of $J_{1}$ and $J_{2}$ would result in φ=0. The general 3-D protraction vector $p^{e}$ is obtained by the difference:

$$p^{e}=v_{12}^{e}-l_{12}u_{s}^{e},$$

where $u_{s}^{e}=R_{s}^{e}{\left[0,0,1\right]}^{T}$ is the head pointing vector, indicated by the red arrow in Figure 2. It is defined to be exactly vertical when the upright posture is assumed during the calibration phase.

## 3. Materials and Methods

### 3.1. Participants

The study was conducted according to the Declaration of Helsinki, juristically verified by the local ethics committee (req-2019-00043), and received written informed consent from all participants. Eight healthy volunteers (four females) were recruited from the university campus. The descriptive characteristics of the participants are presented in Table 1.

### 3.2. Equipment

Neck movements were captured using an optoelectronic VICON^®^ motion capture system (Vicon Motion Systems, Oxford, UK). The neck movements were captured through tripods attached at the middle of the forehead (head), the sternum (sternum), and the midpoint between the cervical vertebras C2 and C7 (neck). The position and orientation of each of these tripods were measured using three reflective markers and rigid bodies of the head and thorax were modeled from their orientation and position data [48]. Raw data were sampled at 120Hz. All signals were used without filtering or elimination of outliers. The angular difference between two tripods was calculated and transformed into Euler angles. Gimbal-lock was prevented by choosing the intermediate Euler angle to consider lateral inclination whose absolute magnitude is guaranteed to be sufficiently different from $90^{\circ }$. For optical verification all measurements were filmed with two VICON Vue^®^ video cameras at 30Hz.

### 3.3. Measurement Procedure

**Figure 3:**
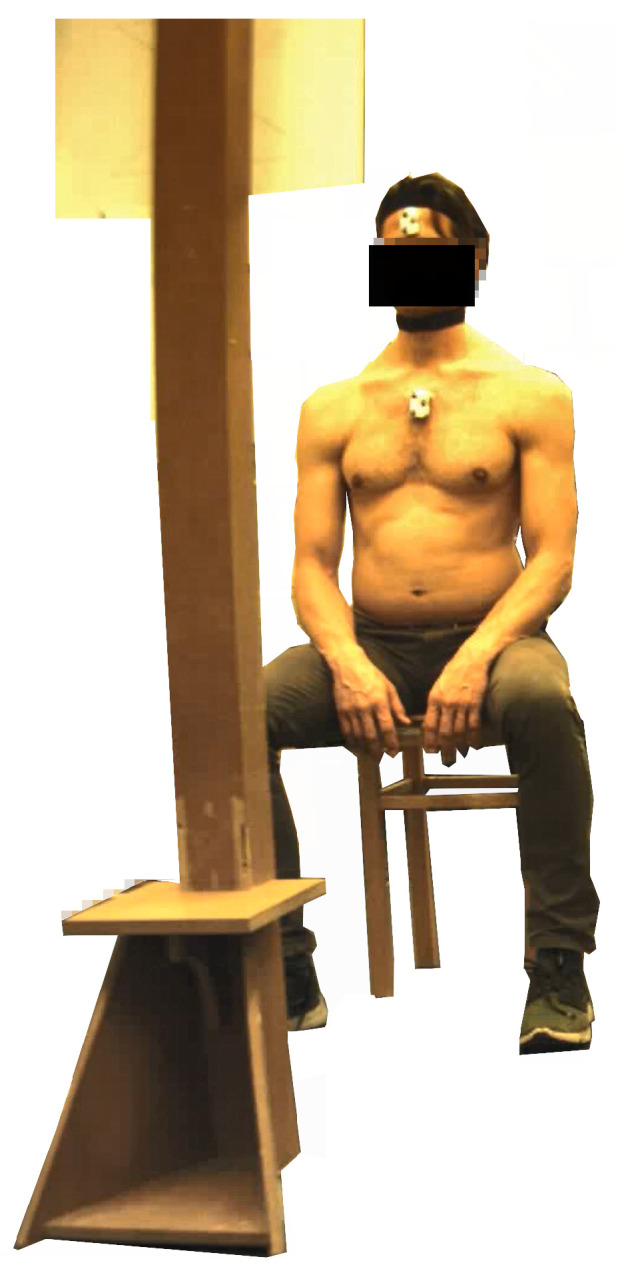
Measurement setup—one participant seated on a stool. The head pose tripoid and the sternum pose tripoid with reflective markers are visible. The neck orientation tripoid is fixated to the black collar. The illustration of the movement pattern is attached to the vertical bar in a way that is clearly visible to the participant.

The length between the vertebrae C7–S2 and C2–C7 was measured following previously described methods [49,50]. Following a static measurement in an upright posture, seated on a stool, each participant performed the tasks described in Table 2. To perform the movement tasks, the participants wore a laser pointer attached to their forehead. The participants were asked to follow the movement patterns as precisely as possible with the laser emitting from the laser pointer. After a practice trial and if necessary manual guidance, two repetitions of the tasks were performed at a self-defined speed in fixed order since any learning or fatigue effects were not of interest for this study. Data from both repetitions were used for further analysis. The measurement setup is illustrated in Figure 3. Participants rested for five seconds between repetitions and one minute between tasks.

### 3.4. Data Analysis

#### 3.4.1. Neck Length Estimates

To estimate the unknown neck length $l_{12}$, the procedure described in Section 2.2 was used. The fitting was conducted individually for each participant (6). Per participant there was a set I of motion patterns i∈I and per motion pattern there was a set of observation time-instants $t\in T_{i}$. The fitting procedure yielded the neck-stick length $l_{12}$ and the constant vectors $v_{s1}$ and $v_{2h}$. Based on these, the head position $o_{h}^{s}$ was predicted from the poses of the sternum and the neck rigid bodies. To verify the model accuracy, the discrepancy between measured and predicted forehead position by evaluating the sum of squared Euclidean errors in (6) was considered and the accuracy of the neck length estimates was expressed as the mean error (ME) and the 5% and 95% percentiles of the error.

#### 3.4.2. Real-World Model Validation

Linear models as described in Section 2 were fitted to describe the dependency between estimated neck-stick length $l_{12}$ versus the measured length C2–C7. The residual errors of the parametrized model for the movement of $J_{2}$ with respect to the fitted model were expressed as ME and the 5% and 95% percentiles of the error.

#### 3.4.3. Noise Sensitivity

Independent identically distributed Gaussian noise samples were added to the position and orientation measurements with a 5- and 10-times higher standard deviation (SD) than is included in the measurements. Using these disturbed noise position measurements, the position and orientation of the thorax, neck, and head were recalculated. From this, the model parameters were identified according to (6). This procedure was repeated with 25 independently generated noise samples for the entire set of measurements taken from a participant and the distribution of the estimated neck length ${\hat{l}}_{12}$ was analyzed. Three variants were considered: (i) Extra position noise only, (ii) extra orientation noise only, and (iii) extra position and orientation noise.

#### 3.4.4. Video Analysis

To further investigate the correlation between neck length C2–C7 and the estimated neck length ${\hat{l}}_{12}$, the participants cervical and thoracic movement patterns were visually investigated from the videos taken during the measurements.

## 4. Results

### 4.1. Neck Length Estimates

Table 3 summarizes the corresponding ME and the 5% and 95% percentiles of $e_{h}$ in three axis for the fitted model. For all participants, the ME is in the lower millimeter range. The error in the predicted position of $J_{2}$ was directly linked to the error of the predicted forehead position via the vector $v_{2h}$, which is a constant when expressed in the *h*-frame. Therefore the model predicts the position $J_{2}$ with an ME in the lower millimeter range.

### 4.2. Real-World Model Validation

**Figure 4:**
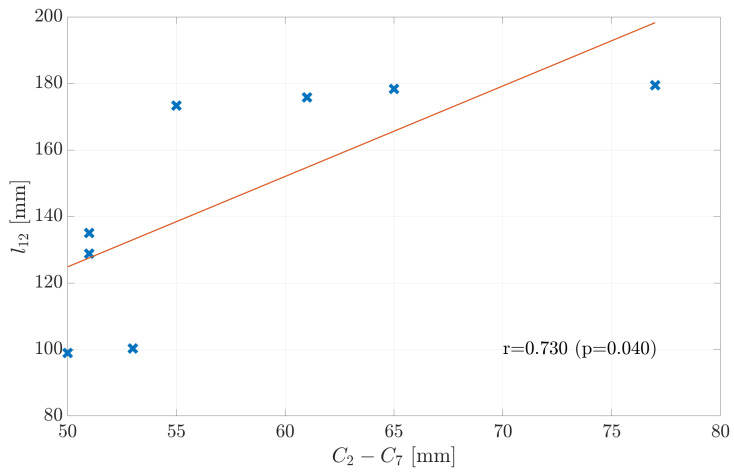
Linear model to describe the fitted length of vector $l_{12}$ depending on the distance between C2 and C7. Data points are labeled with the subject number. The linear model describes the distance $l_{12}$ with a large residual error.

Table 4 and Figure 4 show the residual errors and estimated neck-stick length $l_{12}$ versus the measured length C2-C7 for each participant. To clarify if these residuals are caused by noisy neck length estimates, the impact of extra measurement noise on the estimated neck length ${\hat{l}}_{12}$ in the next section.

### 4.3. Noise Sensitivity

**Figure 5:**
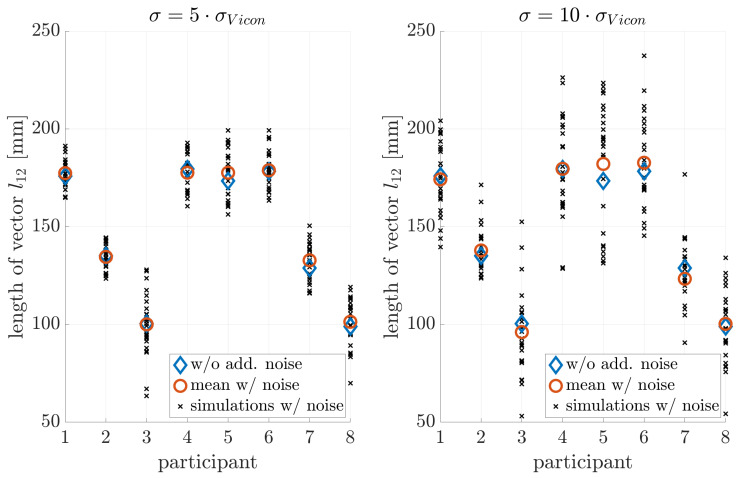
Influence of position noise on the parameter estimation.

**Figure 6:**
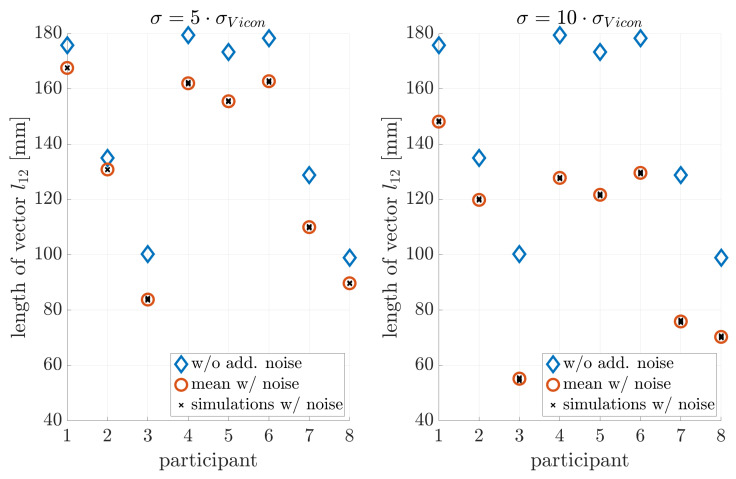
Influence of orientation noise on parameter estimation: A bias towards smaller neck lengths is introduced.

**Figure 7:**
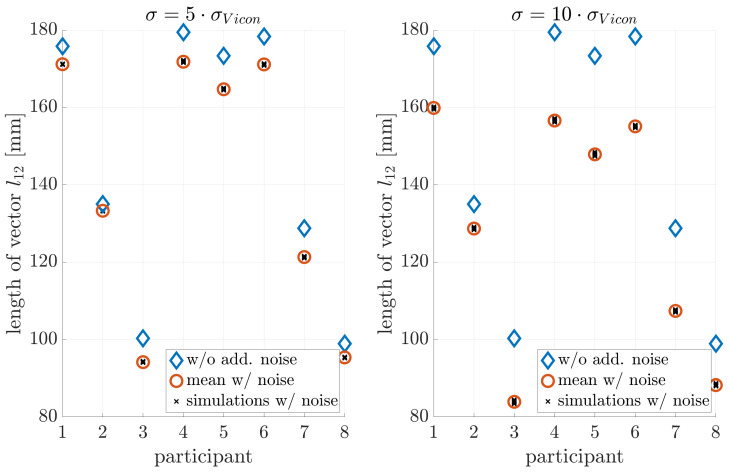
Influence of position and orientation noise on parameter estimation.

Figure 5, Figure 6 and Figure 7 illustrate the noise sensitivity of the neck-length estimator.

The estimation without noise shows a standard deviation of about ±3mm, while orientation noise caused an estimation bias towards shorter neck lengths. This bias was on the order of −1.5 mm if no extra noise was added. Therefore: (i) The measurement equipment and estimation procedure (6) resulted in a neck-length estimate with an uncertainty of few millimeters only. (ii) The effect of noise on the estimation error of ${\hat{l}}_{12}$ was by far too weak to explain the observed residuals on the order of tens of millimeters shown in Figure 4.

### 4.4. Video Analysis

The video analysis revealed varying movement patterns. Participants’ distributed their movement differently over the vertebrae of the neck and upper thorax. Some participants moved with their cervical spine and upper thorax, while others only moved with their cervical spine.

### 4.5. Outlook

In a next step the models quality criteria such as the concurrent validity, reliability, and applicability need to be addressed. The concurrent validity should be assessed by tracking the head position simultaneously with an optoelectronic motion capture system and an IMU system [48]. Reliability should be addressed in a test retest design [37]. Following this, the applicability of the model could be tested in a real life situation, such as during office work.

## 5. Conclusions

The aim of this work was (i) to introduce a simple neck model that describes the relative motion of the head to the thorax and (ii) to measure relative protraction by means of this model and 3D-orientation measurements of the neck and thorax. The model consists of a simple two joint model with joints $J_{1}$ and $J_{2}$ in the vicinity of vertebrae C2 and C7, respectively and a stiff neck-stick of length $l_{12}$ between the joints. The center of joint $J_{2}$ is defined as the head position. The advantage of this model lies in its simplicity, as it describes the head motion with the model parameter $l_{12}$ and the measured orientation of the neck relative to the thorax. The model parameter $l_{12}$ was fitted to the model for different motion exercises. The resulting model describes the relative head motion with a maximum uncertainty of 5 mm only. However, in a practical application the length $l_{12}$ is unknown. To replace its estimation, we propose to predict the length $l_{12}$ from the palpable distance C2–C7, which, however, is a weak predictor. Applying it despite of this, resulted in a protraction measurement error of up to 30% of the true protraction distance in our experiments. It is important to notice, that this error is proportional to the true protraction distance, i.e., the proposed model allows to observe accurate relative protraction. We conclude that the proposed model could be sufficiently accurate to determine protracted malposition in some applications. The advantage of the proposed model is that protraction of the head relative to the thorax can be measured with only two rigid bodies, one on the neck and one on the sternum. This might make it possible to combine this model with more economical measurement methods after it has been validated for these methods.

## Figures and Tables

**Table 1:** Descriptive characteristics of participants.

Participant	Length C7–S2 (cm)	Length C2–C7 (cm)	Height (m)	BMI (kg/m^2^)	Age (Years)
Mean (standard deviation)	50.7 (2.2)	5.8 (0.9)	1.73 (0.09)	24.0 (2.4)	41.1 (11.9)
Participant 1	49.5	6.1	1.61	23.3	30
Participant 2	52.0	5.1	1.81	23.4	52
Participant 3	48.8	5.3	1.65	20.5	31
Participant 4	53.2	7.7	1.81	27.8	28
Participant 5	51.0	5.5	1.72	23.9	52
Participant 6	53.8	6.5	1.87	25.5	53
Participant 7	47.5	5.1	1.69	21.2	31
Participant 8	49.5	5.0	1.68	26.1	52

**Table 2:** Tasks and instructions, except for static sitting and free movement all tasks were repeated twice.

Task	Instructions	Illustration
Static sitting	Sit up straight and straighten up the pelvis. Loosely put your arms on your lap and look straight ahead. Hold this position.	
Zigzag in neutral head position	Follow the zigzag pattern on the wall with your head, keeping as close as possible to the middle line. Start moving to the top left. Repeat this movement three times.	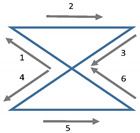
Zigzag in protracted head position	Follow the zigzag pattern on the wall with your head, keeping as close as possible to the middle line. Start moving to the top left. Repeat this movement three times. Hold your head forward.	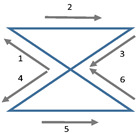
Cross in neutral head position	Follow the cross pattern on the wall with your head, keeping as close as possible to the middle line. Start moving to the top. Repeat this movement three times.	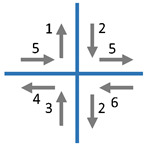
Cross in protracted head position	Follow the cross pattern on the wall with your head, keeping as close as possible to the middle line. Start moving to the top. Hold your head forward. Repeat this movement three times.	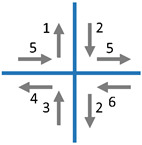
Free movement	Now move your head completely free, at your comfortable speed, for 30 s.	

**Table 3:** Residual errors of the fitted model for the movement of the head; ME—mean error

Participant	ME X [mm]	X Percent. [mm]		ME Y [mm]	Y Percent. [mm]		ME Z [mm]	Z Percent. [mm]	
		5%	95%		5%	95%		5%	95%
1	−0.52	−8.45	3.83	−0.85	−6.96	5.07	1.42	−4.41	8.67
2	−2.62	−10.51	2.83	−0.26	−6.93	5.37	−2.24	−8.36	3.68
3	−1.03	−5.99	3.07	−0.28	−3.95	3.49	−2.48	−7.27	1.92
4	0.98	−3.43	4.88	−3.10	−12.48	1.91	−0.99	−4.86	3.27
5	−3.59	−10.05	2.52	−1.22	−6.74	3.27	1.22	−3.79	6.13
6	−4.96	−11.73	0.71	0.16	−6.15	6.27	2.64	−2.50	7.76
7	−1.34	−5.17	2.39	−2.21	−7.97	6.46	2.41	−3.09	7.31
8	−1.97	−9.76	4.20	−2.81	−9.97	3.84	1.47	−6.31	12.81

**Table 4:** Residual errors of the parametrized model for the movement of $J_{2}$ with respect to the fitted model; ME—mean error.

Participant	ME X [mm]	X Percent. [mm]		ME Y [mm]	Y Percent. [mm]		ME Z [mm]	Z Percent. [mm]	
		5%	95%		5%	95%		5%	95%
1	18.04	15.76	19.51	3.11	2.72	3.59	9.50	6.71	13.20
2	6.75	5.81	7.29	0.21	−0.25	0.70	2.78	1.42	4.61
3	−27.62	−29.29	−25.22	−4.70	−5.83	−3.92	−16.86	−20.34	−14.16
4	−14.11	−16.58	−10.64	−1.82	−2.65	−1.08	−12.57	−15.86	−9.69
5	28.80	25.31	31.27	3.91	2.54	4.83	18.71	14.62	23.67
6	11.20	10.09	11.75	0.64	0.21	0.99	5.22	4.01	7.26
7	0.94	0.82	1.01	−0.03	−0.09	0.03	0.70	0.60	0.84
8	−15.38	−19.73	−9.81	−2.39	−4.55	−1.05	−20.37	−23.91	−16.55

## Data Availability

Data are made available through the corresponding author upon a reasonable request.
